# Modifying the Victor® Easy Set® Rat Trap to Improve the Animal Welfare of Stoats and Ship Rats Trapped in New Zealand

**DOI:** 10.1371/journal.pone.0086760

**Published:** 2014-02-05

**Authors:** Grant A. Morriss, Bruce Warburton

**Affiliations:** Landcare Research, Lincoln, New Zealand; University of Illinois at Urbana-Champaign, United States of America

## Abstract

Stoats (*Mustela erminea*) and ship rats (*Rattus rattus*) in New Zealand are targeted by trapping to mitigate their predation on native wildlife. Internationally recognized guidelines for assessing the effectiveness and welfare performance of kill traps are supported by New Zealand legislation under the Animal Welfare Act 1999. The Victor® Easy Set® rat trap was tested and passed a similar standard for killing short-tailed weasels in Canada but failed for stoats when tested in New Zealand in 2002 (short-tailed weasels and stoats are the same species). We tested a modified version of the trap in 2011–12, modified by changing the treadle trigger to a pull trigger and adding a plastic shroud to direct and align approach by animals to the front of the trap. These traps, in vertical and horizontal sets, were tested with both stoats and ship rats. During each test the trap had to render 10 of 10 animals irreversibly unconscious within 3 minutes to meet approval requirements. The modified trap passed with both species in both trap sets. All stoats were struck across the cranium whereas rats were struck either on the cranium or neck. We recommend this trap design for use by community conservation groups for targeting stoats and ship rats in New Zealand.

## Introduction

European stoats (*Mustela erminea*) were introduced to New Zealand in the mid-1880s to control rabbits (*Oryctolagus cuniculus*) [1]. In recent decades conservationists have recognised the threat that stoats pose to endangered wildlife such as kiwi (*Apteryx* spp.) [2], kaka (*Nestor meridionalis*) [3], [4], yellowhead (*Mohoua ochrocephala*) [3] and blue duck (*Hymenolaimus malacorhynchos*) [5] with many stoat control programmes initiated by state-funded (e.g., the Department of Conservation) and community-led groups. In the early 1970s Fenn traps (MK IV & VI models) were imported to New Zealand from England and became the main tool for monitoring and controlling stoats [6], [7]. Introduced ship rats (*Rattus rattus*) are also targeted in New Zealand as a conservation pest, and because both species are of similar size and can be caught by the same trap types, it was considered important to ensure that any trap developed for stoats could also kill ship rats effectively. Although the target species are pests in New Zealand, there is of course concern about the welfare performance of these traps.

To assess the welfare performance of a kill trap system (including the trap, any boxes or covers used, and the way the trap is set) an animal is monitored while approaching and interacting with a trap, and when caught the time to loss of consciousness and cessation of heartbeat is measured. The International Organisation for Standardisation (ISO) developed a standard for testing traps [8]–[10], and this was then adapted in New Zealand as a National Animal Welfare Advisory Committee (NAWAC) guideline for testing traps [11]. For kill traps to be acceptable, under the NAWAC guidelines, either 10 of 10 or 13 of 15 target animals must be rendered irreversibly unconscious within 3 min of capture. These sample sizes have been selected to minimize the number of animals required per trap test with a 90% probability that traps meet the 3 minute time limit 70% of the time. Unconsciousness is determined by using the palpebral (blinking) reflex, which stops when the animal loses consciousness [12]. The Fenn Mk IV and MkVI traps were tested by Landcare Research in 2001 [13] and eight of nine stoats tested failed to be rendered unconscious within the 3 min required. The Department of Conservation (DOC) was informed of the Fenn trap's poor performance and this led to the development of new kill traps for stoats, ship rats, and ferrets (*Mustela furo*) (i.e., DOC 150, 200 and 250 traps [8], [14], and more recently the Goodnature A24 rat+stoat trap [15]). Although these traps met the NAWAC guideline requirements [16], they are relatively expensive and consequently there is a demand for more economical alternatives.

**Figure 1:**
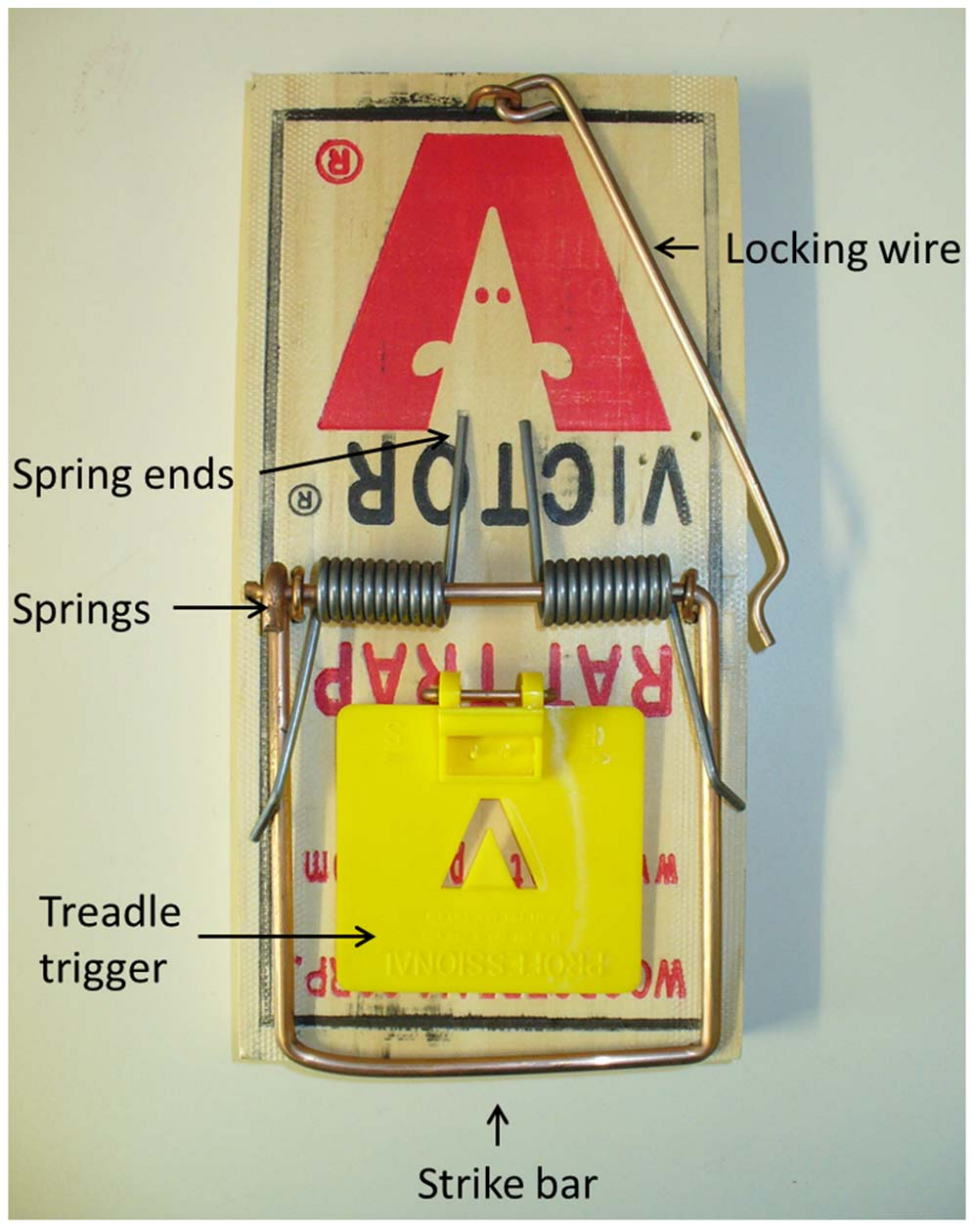
Standard Victor® Easy Set® rat trap.

**Figure 2:**
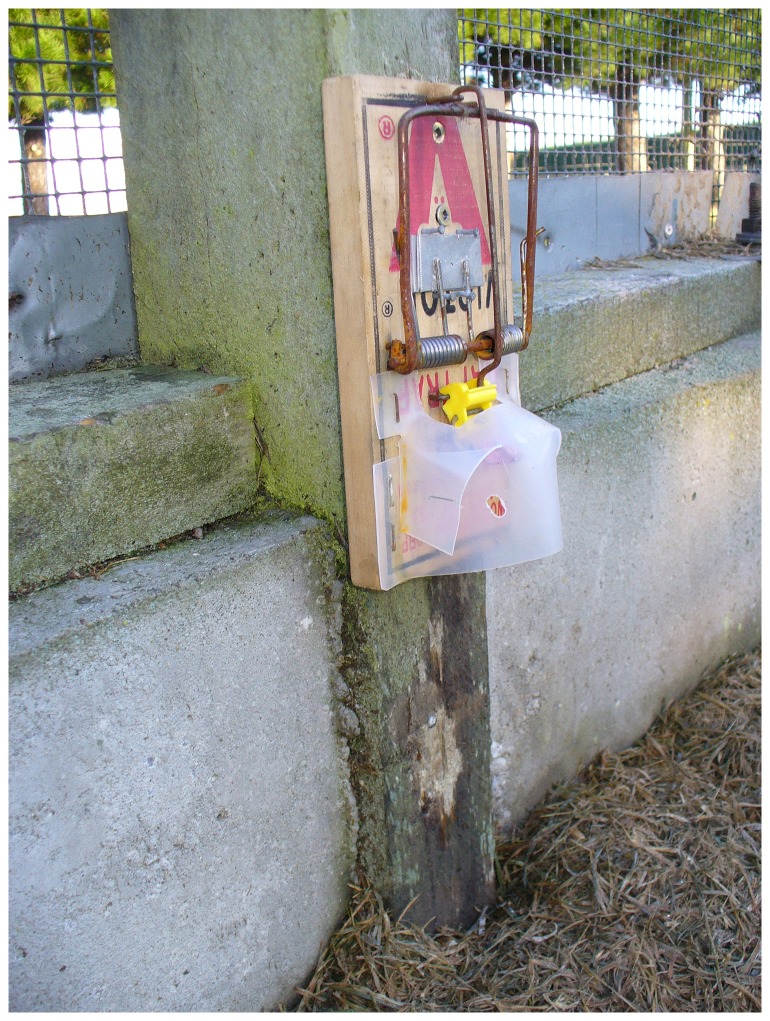
Vertically set modified Victor professional rat trap.

The Victor® Easy Set® rat trap (Figure 1) [17], modified by adding a shroud to achieve more consistent approaches and strike locations (see Figure 2 for an example of a shroud), was tested successfully for killing stoats (referred to as short-tailed weasels, *Mustela erminea*) in Canada [18], and Warburton et al. [19] tested this design on stoats in New Zealand but the trap failed to pass the NAWAC guidelines. Seven stoats were rendered unconscious rapidly (i.e., in <30 s) but an additional three were able to pull out of the trap and escape. The authors concluded that there was insufficient clamping force with this type of trap to hold the animal if they were not rendered unconscious quickly. Although both New Zealand stoats and the Canadian short-tailed weasel are the same species, Canadian animals are generally smaller (maximum recorded weight: females 85 g, males 206 g [20]) than those in New Zealand (maximum recorded weight females: 309 g, males 450 g [19]). Stoats have also been targeted in the UK using modified Victor® rat traps [21] where the modification consisted of the addition of a shroud and of a block placed under the spring-ends purportedly to increase the impact momentum of the trap. Our trial with stoats and ship rats built on this knowledge, with a shroud initially based on the Canadian design, used to increase accuracy and consistency of the strike location and the addition of a spring tensioner with the intention of increasing the impact momentum of the trap. Mechanical testing was undertaken to check whether the spring modification changed the impact momentum as intended.

## Methods

### Ethics Statement

This research was carried out in accordance with the protocol approved by the Landcare Research Animal Ethics Committee (10/01/01).

### Modification to the Victor® Easy Set® Rat Trap

New Victor® Easy Set® traps (Figure 1) were modified by adding: (1) a plastic shroud to the front of the trap (covering the trigger) to direct the stoats' approach so they would be positioned correctly for a cranial strike, (2) a 3-mm-thick spacer under the spring-ends to potentially increase spring tension and therefore impact momentum, and (3) modifying the treadle trigger to a pull trigger. The trigger system and shroud were slightly modified twice after initial tests in an attempt to obtain a consistent strike location (see Results for detail).

### Mechanical Testing of the Modified Victor® Easy Set® Rat Trap

Six new traps were sent to SAI Global (NZ) Ltd to determine the velocity and impact momentum of the striking bar 3 cm above the trap base (i.e. the predicted impact point on a stoat). Three of the traps were modified (with a 3-mm spacer under the spring-ends; Figure 2) and three were unmodified (Figure 1). Each trap was activated three times and the velocity of the strike bar measured with a *Motion*Pro® X3 high speed camera (2000 frames per second). The mean velocity (m/s) and from this the impact momentum (kg.m/s) for each trap was calculated from these three activations. The effective mass of the strike bar was calculated using dimensions measured from the trap and a density for the mild steel of 7850 kg/m^3^. The difference between modified and new traps was compared using a Mann-Whitney U test.

### Pen Tests

Stoats and ship rats from the wild were live-trapped using box and cage traps. Stoats were individually housed in outside cages and had free access to water and a diet of minced rabbit, dead day-old chickens and dry cat food. The ship rats were initially kept in an indoor temperature-controlled room (19±5°C) at the Landcare Research animal facility and housed individually. The rats had free access to water and were fed commercial rodent chow pellets (LabDiet®, Purina Mills) supplemented occasionally with fruit, mixed seeds or dry cat food. This was the standard operating procedure (SOP) for acclimating and husbandry of the two different species (Animal Facility Quality Manual SOP 1∶3, Landcare Research, Lincoln, NZ). Once acclimated, stoats and rats were relocated to one of three outside observation pens (5×5×2 m), with a single trap placed into each. Three individually penned animals were tested at one time. Traps were set at dusk and an observer watched from inside a connecting observation hut for up to 4 h at a time. Once an animal triggered a trap and was captured the observer got to the trap as quickly as possible to measure the time to loss of consciousness (TTLC indicated by the loss of palpebral reflex) by gently touching the eyelid. The TTLC and cessation of heartbeat (measured using a stethoscope) was recorded along with the strike location of the trap on the animal. Captures were also recorded on video using a Bosch Dinion day/night video camera (Bosch Security Systems, Sydney) with a 7–70-mm telephoto lens linked to a Geovision-1248 digital video-recording system (Geovision Incorporated, Taipei). The observation pens were illuminated with infrared light during the hours of darkness.

To pass the test, 10 stoats and 10 ship rats, had to be rendered unconscious within 3 min. If escaped animals survived, they were considered to have exceeded the 3-min time frame to loss of consciousness and therefore had to be counted as a failed capture (i.e. deemed to have been caught but not killed within the required time) [11].

**Figure 3:**
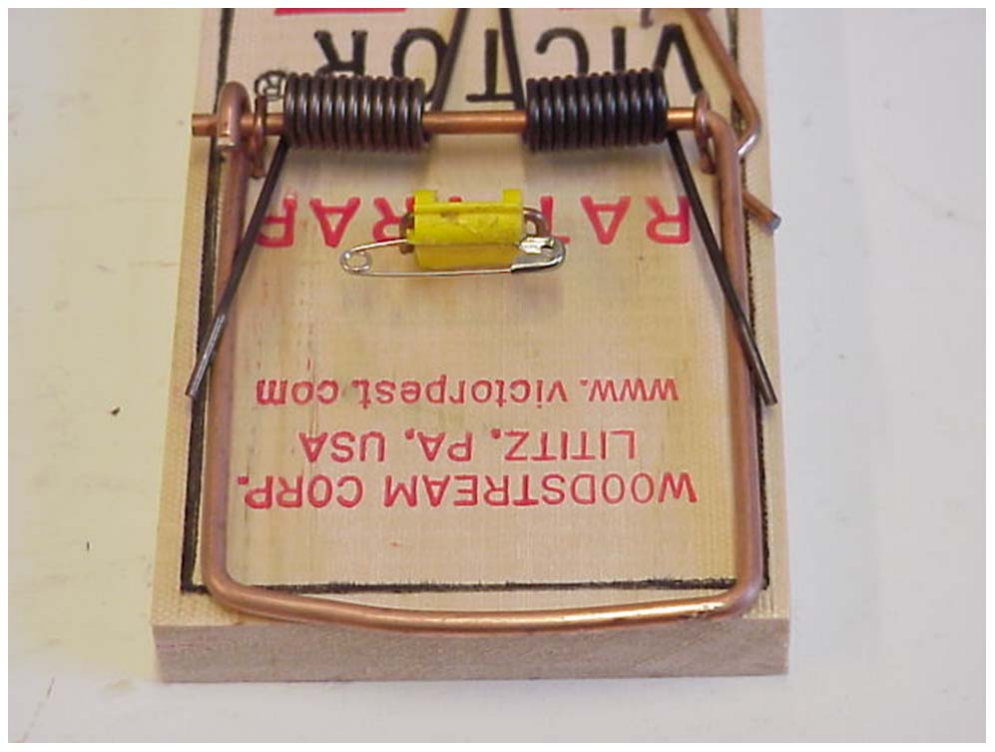
First trigger tested comprising a safety pin through base of the commercially supplied treadle trigger.

**Figure 4:**
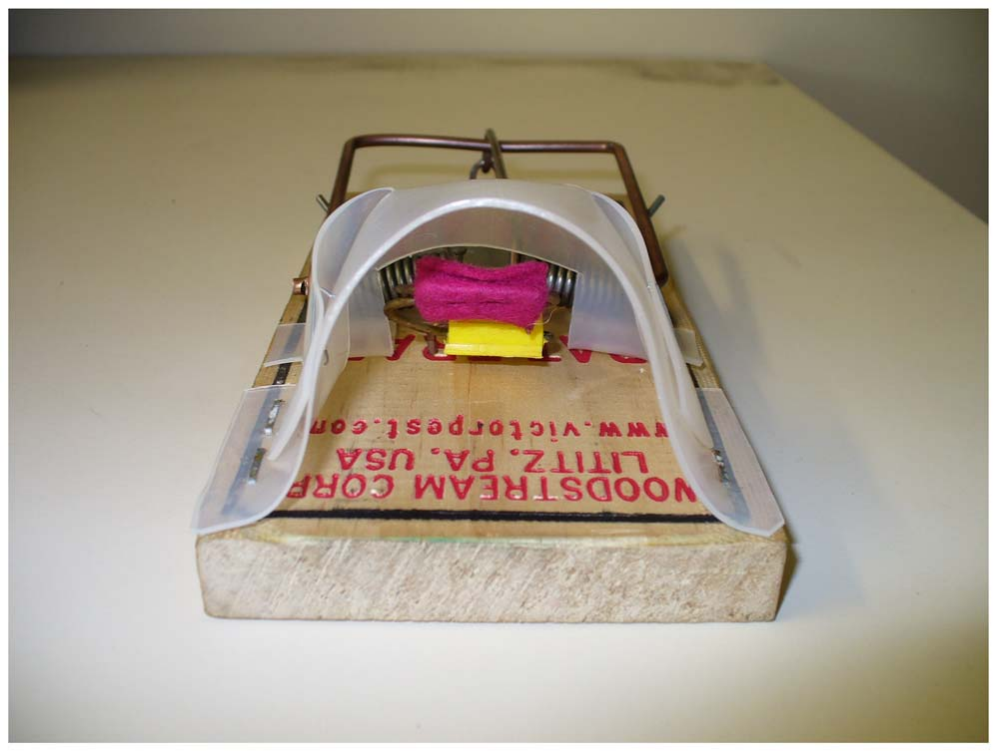
Set trap showing position of the new copper rod and felt trigger.

Two trap set types were tested: (1) Vertical sets: The modified trap was set vertically on a pole 18–20 cm above the ground (Figure 2). This set is an option used to minimize the risk to ground-dwelling birds such as kiwi [19]. The traps were baited with rabbit meat during initial testing on stoats, using a safety pin trigger (Figure 3)—and when the trigger was changed to felt on a wire holder (Figure 4), the felt was soaked in fresh rabbit blood for stoats and smeared with peanut butter for ship rats. Small quantities of rabbit meat or peanut butter were placed on the post beneath the trap to act as an additional lure when trapping stoats or rats respectively.

**Figure 5:**
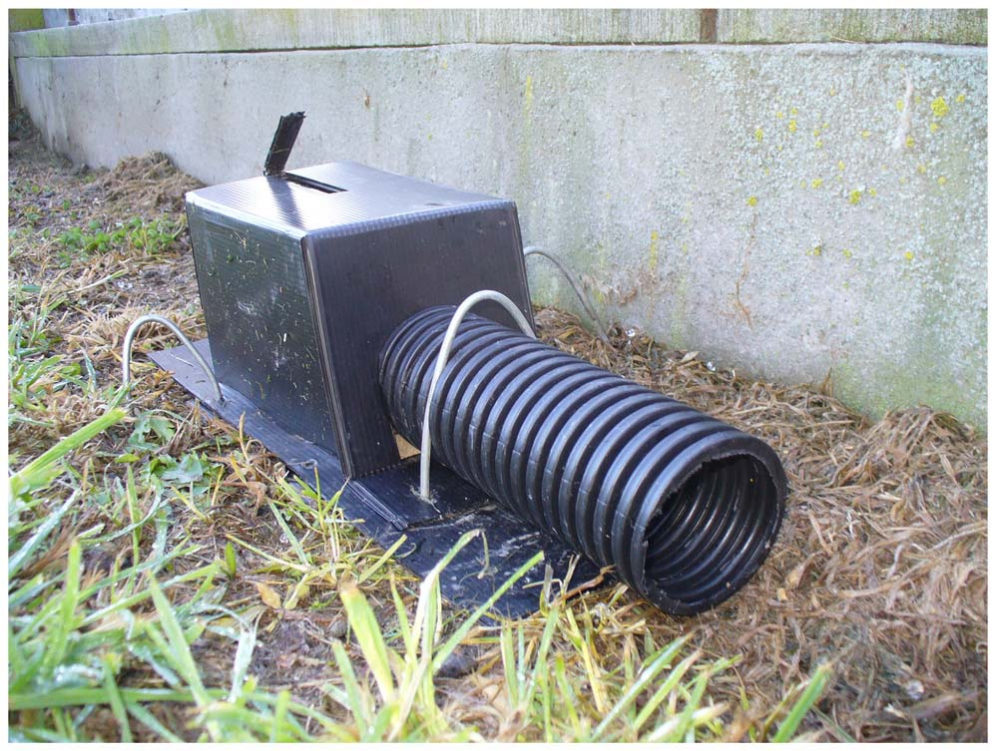
Trap cover for horizontally set Victor professional rat trap. Note slot cut in top of tunnel to allow trigger wire to freely extend upwards when trap is triggered.

(2) Horizontal sets: The modified trap was also set horizontally inside a purpose-built tunnel of lightweight plastic sheeting (180×110×100 mm; Figure 5). A 65-mm-diameter corrugated plastic water pipe (200 mm long) was added to the front of the tunnel to exclude non-target animals larger than stoats or ship rats. The tunnels were held in place with wire pins. The strike location of the second stoat tested in the horizontal set was the nose; this animal was euthanized immediately. The reason for the failure was likely that the locking wire holding the strike bar in the set position had not been able to rise freely when the trap was triggered and thus reduced the momentum of the striking bar, giving the stoat time to back out of the trap slightly. The trap tunnel was modified by cutting a slot in the roof of the tunnel to allow free travel of the locking wire, and testing was restarted.

## Results

### Pen Tests

#### Vertical trap sets for stoats

**Figure 6:**
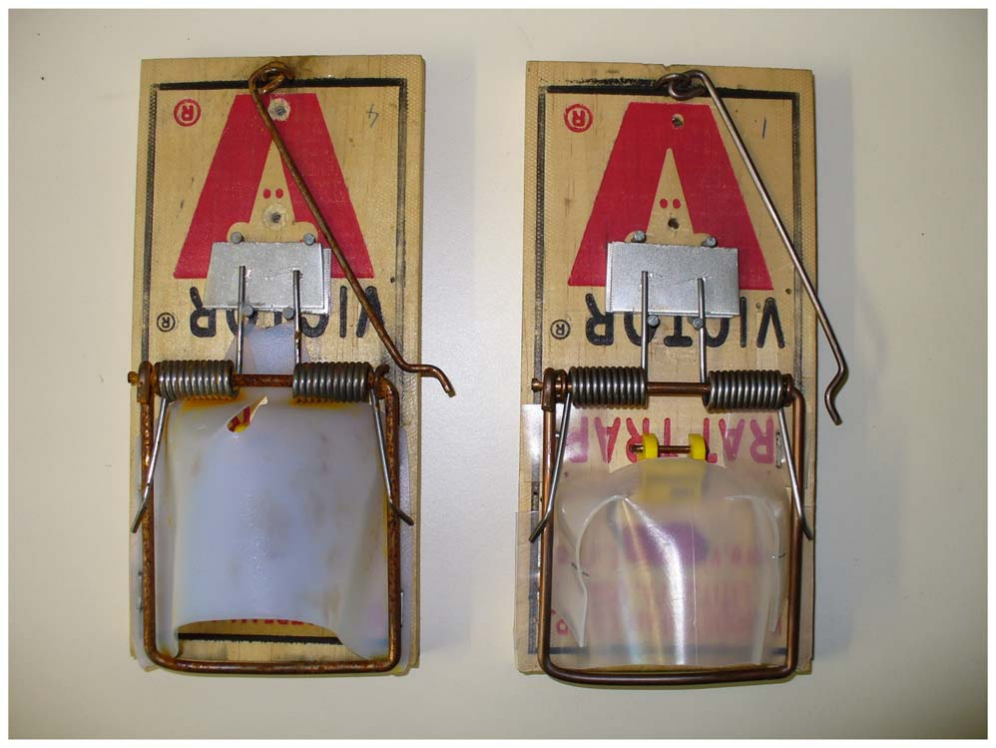
Modified Victor® Easy Set® rat traps with shroud over the trap trigger. The 3-mm-thick spacers are apparent on both traps, obscuring the name ‘Victor’: the first (left) and second (right) modifications.

A total of 22 stoats (13 ♂, 9 ♀) were tested in the vertical trap sets and ranged in weight from 181 g to 376 g. There were four modifications to the original trap before a final test of 10 stoats was successfully completed. Although the first stoat tested was killed successfully (Table 1) the shroud was considered to be too open at the spring end and therefore had potential for a non-lethal strike if a stoat tried to access the baited trigger from the rear. The rear of the shroud was enclosed to reduce the chance of this happening (Figure 6, left). Testing resumed and three stoats were killed successfully before the fourth was struck on the neck and pulled out without sustaining any conspicuous injury (Table 1). This inconsistency of strike location was believed to result from the safety-pin trigger (Figure 3) making it difficult to consistently attach rabbit meat each time the trap was re-baited. A second trigger was therefore developed, which consisted of an s-bend of copper wire (1-mm diameter) covered with felt (Figure 4). The leading edge of the trigger was 30 mm from where the strike bar closed. This resulted in a consistent biting surface when used with liquid lure (i.e., rabbit blood). Testing resumed and a further six stoats were killed successfully with cranial strikes before another stoat was struck on the neck when it pushed in past the trigger. Although stunned by the impact, the stoat pulled out 4 min 40 s after the trap strike (Table 2). The back of the shroud was consequently brought forward by 15 mm to prevent stoats going too far into the trap (Figure 6, right), and then 10 stoats were successfully killed rapidly, all with fractured craniums (Table 2).

**Table 1:** Outcomes of tests of Victor® Easy Set® rat traps set vertically for stoats.

Weight (g)	Sex	Strike location	Palpebral reflex^a^ (min. : s)	Heart stop (min. : s)	Notes
**Test 1:** Rear of shroud open					
252	M	Cranium	<0 : 44	3 : 35	Cranium fractured
**Test 2:** Rear of shroud enclosed					
269	M	Cranium	<0 : 34	3 : 12	Cranium fractured
265	F	Cranium	<0 : 41	3 : 20	Cranium fractured
184	F	Cranium	1 : 13	5 : 32	Hemorrhage at back of cranium with no obvious fracture
376	M	Neck	-	-	Pulled out of trap

A safety pin was attached to the base of the plastic trigger.

a All figures prefixed by ‘<’ indicate that the animal was unconscious when first assessed by the observer.

**Table 2:** Outcomes of tests of Victor® Easy Set® rat traps set vertically for stoats.

Weight (g)	Sex	Strike location	Palpebral reflex^a^ (min. : s)	Heart stop (min. : s)	Notes
**Test 3:** Rear of shroud enclosed					
206	F	Cranium	<0 : 40	3 : 02	Cranium fractured
259	M	Cranium	<0 : 38	2 : 59	Cranium fractured
292	M	Cranium	<0 : 38	3 : 18	Cranium fractured
181	F	Cranium	<0 : 45	3 : 41	Cranium fractured
263	M	Cranium	<0 : 55	5 : 08	Lateral strike
193	F	Cranium	<0 : 35	2 : 48	Cranium fractured
194	F	Neck	-	-	Pulled out of trap at 4 : 40
**Test 4:** Shroud shortened					
273	M	Cranium	<1 : 10	3 : 36	Cranium fractured
338	M	Cranium	<1 : 00	5 : 23	Cranium fractured
285	M	Cranium	<1 : 01	3 : 30	Cranium fractured
205	F	Cranium	<0 : 44	3 : 44	Cranium fractured
224	F	Cranium	<1 : 00	3 : 35	Cranium fractured
242	F	Cranium	<0 : 38	3 : 07	Cranium fractured
323	M	Cranium	<0 : 25	3 : 40	Cranium fractured
363	M	Cranium	<0 : 27	4 : 15	Cranium fractured
285	M	Cranium	<0 : 40	3 : 05	Cranium fractured
317	M	Cranium	<0 : 23	4 : 05	Cranium fractured

Copper wire and felt were attached to the base of the plastic trigger.

a All figures prefixed by ‘<’ indicate that the animal was unconscious when first assessed by the observer.

#### Vertical trap sets for ship rats

Ten rats (3 ♂, 7 ♀), weighing between 98 g and 151 g, were tested using the final modifications to the shroud and trigger. All were killed successfully, with seven struck on the cranium and three struck on the neck (Table 3).

**Table 3:** Outcomes of tests of Victor® Easy Set® rat traps set vertically for ship rats.

Weight (g)	Sex	Strike location	Palpebral reflex^a^ (min. : s)	Heart stop (min. : s)	Notes
101	M	Neck	<1 : 20	3 : 16	
98	F	Neck	<1 : 12	2 : 14	Neck dislocated at C3
110	F	Cranium	<1 : 42	1 : 42	Cranium fractured
109	M	Neck	<1 : 25	1 : 37	
120	F	Cranium	<0 : 49	2 : 06	Cranium fractured
151	F	Cranium	<0 : 45	3 : 10	Cranium fractured
151	F	Cranium	<0 : 48	2 : 10	Cranium fractured
137	F	Cranium	<0 : 32	2 : 39	Cranium fractured
151	M	Cranium	<0 : 31	2 : 02	Cranium fractured
114	F	Cranium	<0 : 28	2 : 37	Cranium fractured

Copper wire and felt were attached to the base of the plastic trigger.

a All figures prefixed by ‘<’ indicate that the animal was unconscious when first assessed by the observer.

#### Horizontal trap sets for stoats

Following the successful test of the vertical set, a total of 12 stoats (5 ♂, 7 ♀), weighing between 181 g and 356 g, were tested in the horizontal sets. The second stoat tested survived when the killing bar struck it in front of the eyes and fractured its sinus bones and was consequently euthanized. The tunnel was consequently modified by cutting a slot in its roof to allow the locking wire to rise freely without impeding the travel of the killing bar (Figure 5). The testing was then restarted with 10 stoats killed successfully (Table 4). Although rendered unconscious quickly, two larger stoats took longer for the heart beat to cease (c. 15–19 min) than observed in prior stoat trap testing.

**Table 4:** Outcomes of tests of Victor® Easy Set® rat traps set in a horizontal tunnel for stoats.

Weight (g)	Sex	Strike location	Palpebral reflex^a^ (min. : s)	Heart stop (min. : s)	Notes
**Test 1:** Tunnel roof intact					
265	M	Cranium	<1 : 32	4 : 08	Cranium fractured
224	F	Nose	-	-	Sinuses fractured
**Test 2:** Tunnel roof slotted to allow free travel of locking rod					
309	F	Cranium	<0 : 40	3 : 58	
227	F	Cranium	<0 : 45	3 : 39	
204	F	Cranium	<0 : 48	4 : 15	
356	M	Cranium	<0 : 52	14 : 55	Pulled out of trap but remained unconscious
206	F	Cranium	<0 : 48	2 : 57	Cranium fractured
308	M	Cranium	<0 : 46	18 : 52	Pulled out of trap but remained unconscious
181	F	Cranium	<0 : 56	3 : 33	
192	F	Cranium	<0 : 32	2 : 48	
301	M	Cranium	<0 : 47	2 : 59	
256	M	Cranium	<0 : 51	3 : 03	

Copper wire and felt were attached to the base of the plastic trigger.

a All figures prefixed by ‘<’ indicate that the animal was unconscious when first assessed by the observer.

#### Horizontal trap sets for ship rats

Following the successful test of the vertical set, 10 ship rats (5 ♂, 5 ♀), weighing between 106 g and 179 g, were tested in the horizontal sets. All rats were killed successfully with 9 of 10 being struck on the cranium by the strike bar (Table 5). The strike location on the 10th rat was inadvertently not recorded but assumed to be the cranium or neck because it was rendered unconscious rapidly.

**Table 5:** Outcomes of tests of Victor® Easy Set® rat traps set in a horizontal tunnel for ship rats.

Weight (g)	Sex	Strike location	Palpebral reflex^a^ (min. : s)	Heart stop (min. : s)	Notes
140	F	Cranium	<0 : 31	2 : 30	
106	F	Cranium	<0 : 34	2 : 46	Cranium fractured
154	M	Cranium	<0 : 35	3 : 20	Cranium fractured
136	F	Cranium	<0 : 26	2 : 48	Cranium fractured
179	M	Cranium	<0 : 36	3 : 32	Cranium fractured
137	M	Cranium	<0 : 34	3 : 17	Cranium fractured
175	M	Cranium	<0 : 28	2 : 37	Cranium fractured
162	M	Cranium	<0 : 37	2 : 24	Cranium fractured
166	F	Cranium	<0 : 46	2 : 28	Cranium fractured
107	F	Cranium	<0 : 41	2 : 17	Cranium fractured

Copper wire and felt were attached to the base of the plastic trigger.

a All figures prefixed by ‘<’ indicate that the animal was unconscious when first assessed by the observer.

#### Mechanical testing of the modified Victor® Easy Set® rat trap

There was no significant difference in impact momentum (*p* = 0.126) between modified (mean = 0.323 kg.m/s) and unmodified traps (mean = 0.338 kg.m/s). The velocity (averaged across three traps each with three firings measured at the anticipated point of impact) of the modified and standard traps was 27.86 m/s and 29.10 m/s respectively which was not significantly different (*p* = 0.126).

## Discussion

The modifications used in this study enabled the Victor® Easy Set® rat trap to accurately and consistently target the cranium and render stoats and ship rats irreversibly unconscious rapidly. Though the measurements of irreversible unconsciousness varied, in reality this was due to the time it took the observer to reach the trapped animal and check the palpebral reflex, rather than any variation in unconsciousness exhibited by the animals. Most, if not all, of those successfully trapped animals would have become unconscious in less than 30 s given the level of cranial trauma caused by the trap. Although the NAWAC trap testing guidelines allow kill traps to pass even with times to irreversible unconsciousness of up to 3 minutes, those traps that render animals irreversibly unconscious much quicker should be promoted to maximize animal welfare outcomes. This ideal outcome is being met by the Victor® Easy Set® rat trap along with the other kill trap models (DOC series and Goodnature A24) commonly used to target stoats.

The addition of a small block placed under the spring-ends did not increase the impact momentum as initially expected, so did not significantly increase striking bar velocity. Results of the past trials are equivocal because a 410-g male stoat survived a cranium strike with the standard spring tension trap whereas a 450 g male was killed quickly [19]. In these current trials the two large stoats (308 g and 356 g) that had prolonged heart stop times in the horizontal test may indicate that the modified trap is only just adequate for killing New Zealand stoats and to address this potential weakness would require stronger springs that can deliver greater impact momentum. Some of this variability in apparent killing effectiveness might be a result of variability in impact momentum between individual traps although for these traps the coefficient of variation was only 3.8%. Such variability is similar to that reported for a series of rotating-jaw conibear traps for which the impact momentum varies from only 4 to 7% of the mean [22].

It seems that a factor critical to achieving effective stoat kills is to consistently strike the cranium. We found that strikes on the neck of stoats (with little subsequent clamping force) did not kill them quickly (our initial testing with the vertically set traps resulted in two neck strikes that failed to kill the animals and enabled them to escape). Consequently the shape and size of the shroud and positioning of the bait is critical to ensure that the killing bar strikes the cranium consistently. Wherever possible, the number of animals used in trap testing should be minimized. Ideally, testing would be based on mechanical tests of trap performance, in conjunction with computer simulation or animal analogues [23], [24]. However, it is vital that the effect of the target animal's behavior is not overlooked. That is, for traps that rely on accurate strike locations, especially those with a single striking bar, it is essential that additional factors such as distance between bait and strike bar, shroud design, and animal position and potential variability in this are taken into account. As Warburton and Hall [9] and Baker et al. [24] suggest, mechanical tests could be used as a first step to determine if a trap has the potential to kill quickly before testing progresses to using live animals.

For the modified trap design it will be necessary to use a liquid or paste lure that can be soaked into or smeared onto the felt trigger. This will ensure the bite of the target animals on the trigger is consistent. The lure used in the pen trials was fresh rabbit blood, which has a limited field life depending on ambient temperature. In winter fresh blood may remain attractive for up to a week whereas in warmer months it is likely to last only 2 or 3 days at best. Work is required to develop a synthetic lure that can be incorporated into a slow-release material to replace the felt and provide a long-life attractant.

The Victor® Easy Set® trap with the tested modification provides a low-cost, light-weight, humane trapping option for control of stoats and ship rats. The two setting options (vertical sets and horizontal tunnel sets) will minimize the risk to non-target species especially threatened indigenous kiwi and weka (*Gallirallus australis*). These trials have only tested the killing and welfare performance of the traps, and field trials are now required to test their capture efficiency and compare these traps with the more expensive alternatives.
